# Effects of Sex, Age, and Fasting Conditions on Plasma Lipidomic Profiles of Fasted Sprague-Dawley Rats

**DOI:** 10.1371/journal.pone.0112266

**Published:** 2014-11-06

**Authors:** Kosuke Saito, Masaki Ishikawa, Mayumi Murayama, Masayo Urata, Yuya Senoo, Katsuko Toyoshima, Yuji Kumagai, Keiko Maekawa, Yoshiro Saito

**Affiliations:** 1 Division of Medical Safety Science, National Institute of Health Sciences, 1-18-1 Kamiyoga, Setagaya, Tokyo, Japan; 2 Clinical Research Center, Kitasato University School of Medicine, 1-15-1 Kitasato, Minami, Sagamihara, Kanagawa, Japan; University of California, Los Angeles, United States of America

## Abstract

Circulating lipid molecules reflect biological processes in the body and, thus, are useful tools for preclinical estimation of the efficacy and safety of newly developed drugs. However, background information on profiles of circulating lipid molecules in preclinical animal models is limited. Therefore, we examined the effects of multiple factors such as sex (fasted male vs. female), age (fasted 10 vs. 30 weeks old), and feeding conditions (feeding vs. fasting, 16 vs. 22 hr fasting, 10 AM vs. 4 PM blood collection), on the global profiles of lipid molecules in plasma from Sprague-Dawley rats by using a lipidomic approach. Our assay platform determined 262 lipid molecules (68 phospholipids, 20 sphingolipids, 138 neutral lipids, and 36 polyunsaturated fatty acids and their metabolites) in rat plasma. Multivariate discriminant analysis (orthogonal partial least squares discriminant analysis) and heat maps of statistically significant lipid molecules revealed that the plasma lipid profiles in rats are predominantly influenced by feeding conditions, followed by sex and age. In addition, the fasting duration (16 vs. 22 hr fasting) or the time of blood collection (10 AM vs. 4 PM blood collection) has limited or no contribution on the profiles of lipid molecules in rat plasma. Our results provide useful, fundamental information for exploring and validating biomarkers in future preclinical studies and may help to establish regulatory standards for such studies.

## Introduction

Circulating metabolites are useful tools to diagnose diseases such as lung and gastrointestinal cancer [1]–[3]. The advantages of diagnostic applications using circulating metabolites are non- or low-invasiveness, as well as homogeneity between humans and experimental animal models. These advantages are also shared with the application of circulating metabolites as biomarkers for estimation of the efficacy and safety of newly developed drugs. The rat is a useful and essential animal model for preclinical studies investigating drug metabolism, pharmacokinetics, and toxicological tests [4], [5]. Therefore, background data of circulating metabolites in rats are useful in designing such studies.

Lipids, including fatty acids and their metabolites, phospholipids, sphingolipids, and neutral lipids, are one major class of circulating metabolites, and their hydrophobicity could allow their levels in blood to substantially reflect their levels in tissues (e.g., liver and brain tissues) [6], [7]. Phospholipids and sphingolipids are major components of cell membranes, and neutral lipids serve as energy sources for the cells. The phospholipid 18∶0/18∶1 phosphatidylcholine (PC) acts as a circulating regulator of fatty acid uptake in muscle [8], and ceramides (Cer), a class of sphingolipids, mediate saturated fatty acid-induced insulin resistance [9]. Fatty acid metabolites such as arachidonate metabolites are signaling molecules of inflammatory response [10]. Therefore, lipids are potential biomarkers to predict the efficacy and toxicity of newly developed drugs.

Circulating lipids in human have been reported to vary with blood matrices and subjects’ backgrounds, including sex and age, as well as sample collection and storage conditions. Our previous reports demonstrated that plasma and serum, two matrices obtained from blood, presented different profiles of circulating lipids [11], [12]. For example, the levels of more than 100 lipid molecules showed differences between plasma and serum samples from aged women [12]. Sphingolipids are present at higher concentration in blood in female than in male individuals [12]–[14]. Storage temperature and freezing-thawing cycles affect the levels of many lipid species, including diacylglycerols (DGs) [12], [15]. In addition, of 80 lipids circulating in the plasma, majorities were present at varying levels among different anticoagulant supplements (citrate, EDTA, and heparin) [16]. However, few comprehensive studies focusing on the factors affecting circulating lipids have been performed in preclinical animal models such as rats.

A lipidomic approach is a high-throughput measurement of a broad range of lipid molecules [17], [18]. This method applies liquid chromatography for the separation of lipid molecules and mass spectrometry for their qualitative and quantitative analyses. Although the range of measurable lipid classes varies among assay platforms, a lipidomic approach usually can measure over 100 lipid molecules from plasma as well as from tissues (e.g., liver tissues) [19], [20]. Recently, we characterized and measured 253 lipid molecules in our assay platforms by using human blood plasma [12]. In the present study, we employed the same platforms and examined the effects of multiple factors on circulating lipids, including sex, age, and feeding conditions (feeding, length of fasting period, and diurnal time of sample collection) in the plasma of rats, which is the most-frequently used animal model in preclinical studies. In total, we determined and examined 262 lipid molecules (68 phospholipids, 20 sphingolipids, 138 neutral lipids, and 36 polyunsaturated fatty acids [PUFAs] and their metabolites). Multivariate statistical analysis, i.e., orthogonal partial least squares discriminant analysis (OPLS-DA), demonstrated that the plasma lipid profiles of rats are predominantly affected by feeding conditions, followed by sex and age. No component separating length of fasting period or diurnal time of sample collection was observed. In addition, we also addressed the effects of multiple factors on individual circulating lipid molecules.

## Materials and Methods

### Animals

**Figure 1:**
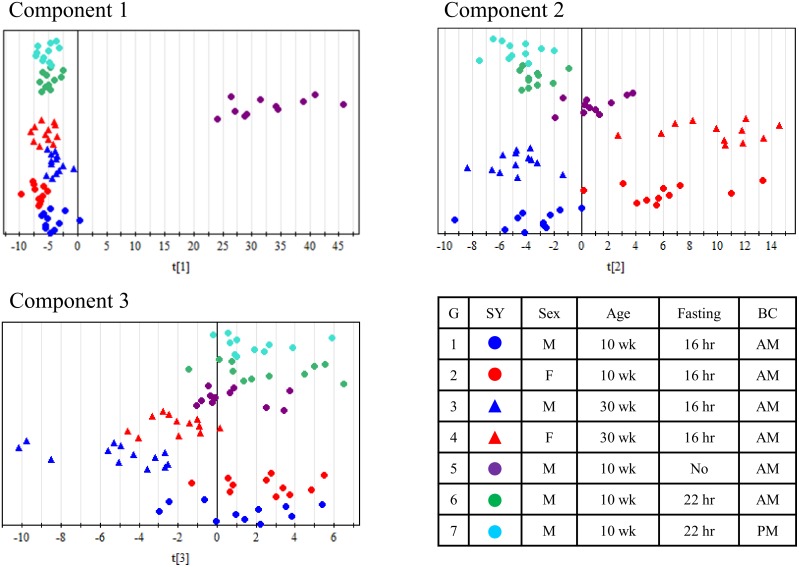
OPLS-DA model of overall profiles of lipid molecules. The goodness-of-fit parameters R2X, R2Y, and Q2 values are as follows; 0.469, 0.159, and 0.149 for component 1, 0.094, 0.143, and 0.127 for component 2, and 0.034, 0.107, and 0.088 for component 3. M, male; F, female; wk, week; SY, symbol; BC, time of blood collection.

Male and female Sprague-Dawley rats (8 weeks old) were purchased from Charles River Japan (Kanagawa, Japan) and housed until they were 10 or 30 weeks old. The animals were housed in a 12-hr light/dark cycle and were allowed food (CRF-1, nutrient composition were described in Table S1; Oriental Yeast, Tokyo, Japan) and water ad libitum. The plasma samples were obtained in the presence of EDTA-Na from rats after the indicated fasting periods and were collected at the indicated times (Fig. 1A and Table S2). The plasma samples were frozen immediately and stored at −80°C. The use of animal specimens was approved by the Ethics Review Committee for Animal Experimentation of the National Institute of Health Sciences (Tokyo, Japan).

### Lipid extraction

Lipid extraction was performed as described previously [12]. In brief, lipids were extracted from 90 µL of plasma by using the Bligh and Dyer’s method with a few modifications. The lower organic phase was used for measurement of phospholipids, sphingolipids, and neutral lipids. The upper aqueous phase was subjected to solid extraction and was used for measurement of PUFAs and their metabolites. Hexadeuterated 16∶0/16∶0 PC (16∶0/16∶0-d6 PC; Larodan Fine Chemicals, Malmo, Sweden) and tetradeuterated leukotriene B4 (LTB4-d4; Cayman Chemical, Ann Arbor, MI) were added as internal standards before extraction.

### Measurement of phospholipids, sphingolipids, and neutral lipids

Phospholipids, sphingolipids, and neutral lipids were measured using liquid chromatography-time-of-flight mass spectrometry (LC-TOFMS; ACQUITY UPLC System [Waters, Milford]-LCT Premier XE [Waters, Milford]), as described previously [12]. The samples from each experimental group were randomized across the run. Raw data obtained by LC-TOFMS were processed using the 2DICAL software (Mitsui Knowledge Industry, Tokyo, Japan), which allows detection and alignment of the ion peaks of each ionized biomolecule obtained at the specific m/z and column retention time (RT). The main parameter of 2DICAL was set as described previously with a few modifications [12]. To extract the ion peaks of phospholipids (lysophosphatidylcholine [LPC], lysophosphatidylethanolamine [LPE], PC, ether-type PC [ePC], PE, ePE, phosphatidylinositol [PI]) and sphingolipids (sphingomyelin [SM], Cer, hexosylceramide [HexCer]), the RT range was from 2.0 to 38.0 min in the negative ion mode; while for ion peaks of neutral lipids (cholesterol/cholesterol ester [Ch/ChE], DG, triacylglycerol [TG], coenzyme Q), the RT range was from 2.0 to 60.0 min in the positive ion mode. The intensities of each extracted ion peak were normalized to those of the internal standard (16∶0/16∶0-d6 PC). To monitor experimental quality throughout extraction, measurement, and data extraction, the relative standard deviation (RSD) of the internal standard (16∶0/16∶0-d6 PC) was calculated (7.34%). Extracted ion peaks were subjected to identification of lipid molecules by comparison of the ion features, including RT, m/z, preferred adducts, and in-source fragments, of the experimental samples with those of our reference library of lipid molecule entries, as described previously [12]. Processing of extracted ion peaks yielded 226 lipid molecules (88 and 138 lipid molecules from negative ion mode and positive ion mode, respectively; Table 1 and Table S3).

**Table 1:** Lipid classes identified and numbers of individual lipid molecules in rat plasma.

Lipid types	Detected ionmode	Lipid classes	Number of molecules
		lysophosphatidylcholine (LPC)	9
		lysophosphatidylethanolamine (LPE)	1
		phosphatidylcholine (PC)	40
Phospholipid	Negative	ether-type PC (ePC)	4
		phosphatidylethanolamine (PE)	4
		ether-type PE (ePE)	3
		phosphatidylinositol (PI)	7
		sphingomyelin (SM)	14
Sphingolipid	Negative	ceramide (Cer)	5
		hexosylceramide (HexCer)	1
		cholesterol/cholesterolester (Ch/ChE)	26
Neutral lipid	Positive	diacylglycerol (DG)	10
		triacylglycerol (TG)	101
		coenzyme Q (CoQ)	1
poly unsaturatedfatty		arachidonic acid (AA) and its metabolites	18
acids (PUFAs)	Negative	eicosapentaenoic acid (EPA) and itsmetabolites	8
and theirmetabolites		docosahexaenoic acid (DHA) and itsmetabolites	10
		total	262

### Measurements of PUFAs and their metabolites

PUFAs and their metabolites were measured by targeted approach using LC-MS/MS (ACQUITY UPLC System-5500QTRAP quadrupole-linear ion trap hybrid mass spectrometer [AB Sciex, Framingham, MA]), as described previously [12]. The samples from each experimental group were randomized across the run. Targeted lipid molecules were annotated by comparison of RT, parent ion, and MS/MS ion fragments with standard lipid molecules using MultiQuant Software (Version 2.1, AB Sciex). The intensities of each ion peak from targeted lipid molecules were normalized to those of the internal standard (LTB4-d4). To monitor experimental quality throughout extraction, measurement, and data processing, the RSD of the internal standard (LTB4-d4) was calculated (18.22%). Processing of targeted lipid molecules yielded 36 lipid molecules (Table 1 and Table S3).

### Data processing

All data obtained of individual lipid molecules were normalized to the median values of all measured samples as 1. The average values ± standard deviations of the normalized levels of each lipid molecule in each study group are presented in Table S4. For overall comparison, data were loaded into SIMCA-P+12 (Umetrics, Umea, Sweden), pareto-scaled, and analyzed using OPLS-DA to visualize the variance among the groups in the present study. Comparison of individual metabolite levels among groups was performed by the Welch’s t-test to assess statistical differences. In the present study, p<0.05 represents statistical significance.

## Results

### Effects of multiple factors on global profiles of lipid molecules in rat plasma

To compare the differences caused by multiple factors (sex, age, and feeding conditions) on the determined 262 lipid molecules, the OPLS-DA model was applied. In the present study, we employed fasted conditions to examine the effect of sexes and ages, because food intake affects lipidomic profiles and therefore fasted condition is prefer to identify biomarkers from lipid molecules in preclinical study. We used all 7 groups listed in Figure 1 and Table S2. As shown in Fig. 1, component 1 separated fed (purple circle) and fasted (all others) rats; component 2 separated female (red) and male (blue, purple, green, and light blue) rats,; and component 3 separated 10-week-old (dot) and 30-week-old (triangle) rats. There is no component separating fasting duration (16 hr [blue dot] vs. 22 [green dot]) or time of blood collection (10 AM [green dot] vs. 4 PM [light blue dot]).

### Differences in the fasted levels of lipid molecules between sexes

**Figure 2:**
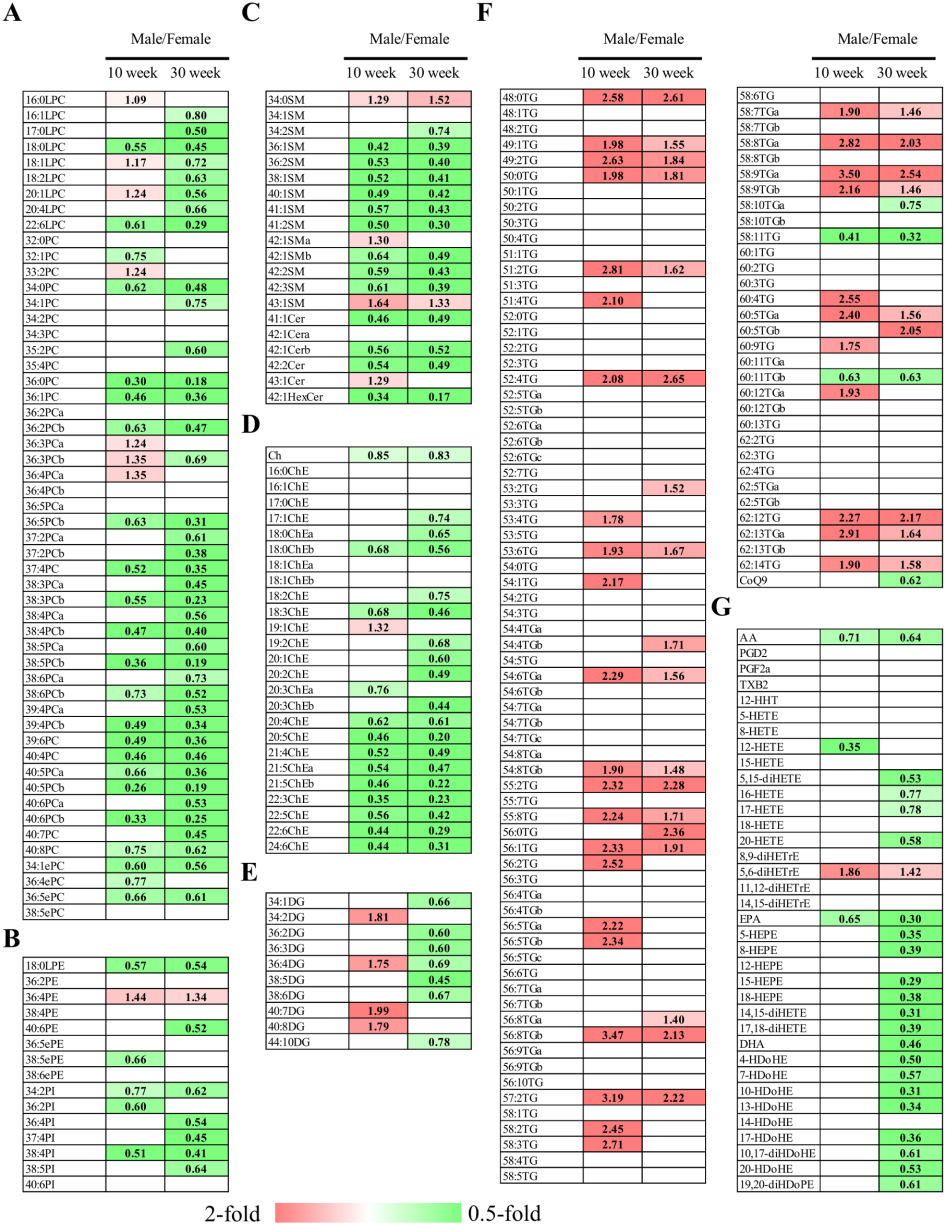
Heat maps of lipid molecules among fasted male and female rats. The heat maps were generated with statistically significant different lipid molecules (p<0.05) of lysophosphatidylcholines and phosphoatidylcholines (A), other phospholipids (B), sphingolipids (C), cholesterol/cholesterolesters (D), diacylglycerols (E), triacylglycerol and coenzyme Q9 (F), and arachidonic acid, eicosapentaenoic acid, docosahexaenoic acid, and their metabolites (G). The numbers indicate the fold change of levels of individual lipid molecules in male over female at 10 and 30 weeks old. The alphabet of the lipid class is used to distinguish 2 lipid species possessing the same formula. LPC, lysophosphatidylcholine; PC, phosphatidylcholine; ePC, ether-type PC; LPE, lysophosphatidylethanolamine; PE, phosphatidylethanolamine; ePE, ether-type PE; SM, sphingomyelin; Cer, ceramide; HexCer, hexosylceramide; Ch, cholesterol; ChE, cholesterolester; DG, diacylglycerol; TG, triacylglycerol; CoQ9, coenzyme Q9; AA, arachidonic acid; PG, prostaglandin; TX, thromboxane; HETE, hydroxyeicosatetraenoic aicd; diHETE, dihydroxyeicosatetraenoic acid; diHETrE, dihydroxyeicosatrienoic acid; EPA, eicosapentaenoic acid; HEPE, hydroxyeicosapentaenoic acid; DHA, docosahexaenoic acid; HDoHE, hydroxydocosahexaenoic acid; diHDoHE, dihydroxydocosahexaenoic acid; diHDoPE, dihydroxydocosapentaenoic acid.

To gain insight into sex-dependent differences in plasma lipid molecules, we next compared the levels of individual lipid molecules between fasted male and female rats. Of the 262 lipid molecules we determined, 110 and 142 lipid molecules were significantly different between male and female at 10 and 30 weeks old, respectively (Table 2). Of these lipid molecules, 67 lipid metabolites were commonly changed among 10- and 30-week-old rats (Fig. 2). Higher levels of most of the SMs (9/14 molecules), such as 36∶1 and 36∶2 SM, were consistent among 10- and 30-week-old female rats (Table 2 and Fig. 2C). In addition to the SMs, PUFAs containing ChEs are higher in both 10- and 30-week-old female rats (Fig. 2D). On the other hand, higher levels of PUFAs and their metabolites, such as eicosapentaenoic acid (EPA) and 5-hydroxyeicosatetraenoic acid, were almost specific for 30-week-old female rats. Furthermore, most of the PUFAs and their metabolites that showed higher levels in 30-week-old female rats are EPA, docosahexaenoic acid (DHA), and their metabolites (Fig. 2G).

**Table 2:** Sex-based differences in the number of lipid molecules at significantly higher levels.

		10 week		30 week		Common	
Lipid classes	Total lipid molecules	Male	Female	Male	Female	Male	Female
Phospholipids	68	8	28	1	46	1	24
Sphingolipids	20	4	13	2	14	2	13
Neutral lipids	138	38	15	27	30	22	2
PUFAs and metabolites	36	1	3	1	21	1	2
Total	262	51	59	31	111	26	41

### Differences in the fasted levels of lipid molecules between ages

**Figure 3:**
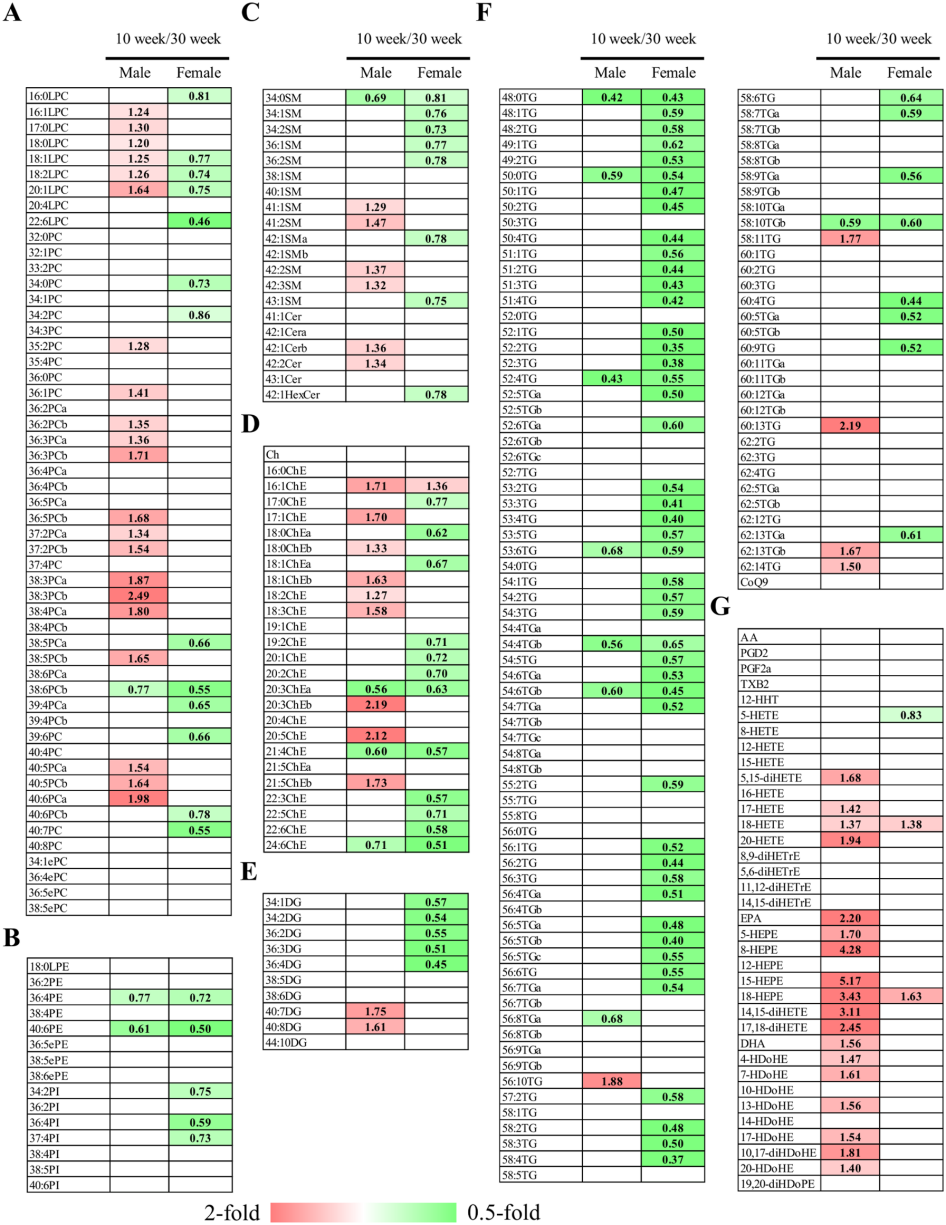
Heat maps of lipid molecules among fasted rats of different age groups. The heat maps were generated with statistically significant different lipid molecules (p<0.05) of lysophosphatidylcholines and phosphoatidylcholines (A), other phospholipids (B), sphingolipids (C), cholesterol/cholesterolesters (D), diacylglycerols (E), triacylglycerol and coenzyme Q9 (F), and arachidonic acid, eicosapentaenoic acid, docosahexaenoic acid, and their metabolites (G). The numbers indicate the fold change of levels of individual lipid molecules in 10-week-old over 30-week-old male and female rats. The alphabet of the lipid class is used to distinguish the 2 lipid species possessing the same formula. LPC, lysophosphatidylcholine; PC, phosphatidylcholine; ePC, ether-type PC; LPE, lysophosphatidylethanolamine; PE, phosphatidylethanolamine; ePE, ether-type PE; SM, sphingomyelin; Cer, ceramide; HexCer, hexosylceramide; Ch, cholesterol; ChE, cholesterolester; DG, diacylglycerol; TG, triacylglycerol; CoQ9, coenzyme Q9; AA, arachidonic acid; PG, prostaglandin; TX, thromboxane; HETE, hydroxyeicosatetraenoic aicd; diHETE, dihydroxyeicosatetraenoic acid; diHETrE, dihydroxyeicosatrienoic acid; EPA, eicosapentaenoic acid; HEPE, hydroxyeicosapentaenoic acid; DHA, docosahexaenoic acid; HDoHE, hydroxydocosahexaenoic acid; diHDoHE, dihydroxydocosahexaenoic acid; diHDoPE, dihydroxydocosapentaenoic acid.

Subsequently, we compared the levels of individual lipid molecules between 10- and 30-week-old rats. In fasted male rats, 76 lipid molecules were significantly different, and the levels of 61 molecules were high in 10-week-old rats whereas the levels of 15 molecules were high in 30-week-old rats (Table 3). The phospholipids that presented higher levels at 10 weeks in male rats were LPCs and PCs (Fig. 3A and B). In addition, most of the PUFAs and their metabolites that showed higher levels at 10 weeks in male rats than those at 30 weeks were EPA, DHA, and their metabolites (Fig. 3G). In contrast, in fasted female rats, 101 lipid molecules were significantly different, and the levels of 98 were higher at 30 weeks than at 10 weeks (Table 3). Most of these lipid molecules are neutral lipids (72 out of 101). As shown in Fig. 3F, levels of relatively shorter and less unsaturated TGs, such as 48∶0, 50∶0, and 52∶1 TGs, were higher at 30 weeks than at 10 weeks in female rats. In contrast, levels of relatively longer and highly unsaturated TGs, such as 56∶10, 58∶11, and 60∶13 TGs, were lower at 30 weeks than at 10 weeks in male rats. Only 16 lipid molecules, such as 36∶4 and 40∶6 PE, showed significantly different levels between 10- and 30-week-olds in both male and female rats (Fig. 3).

**Table 3:** Age-based differences in the number of lipid molecules at significantly higher levels.

		Male		Female		Common	
Lipid classes	Total lipidmolecules	10 week	30 week	10 week	30 week	10 week	30 week
Phospholipids	68	21	3	0	18	0	3
Sphingolipids	20	6	1	0	8	0	1
Neutral lipids	138	16	11	1	71	1	10
PUFAs and metabolites	36	18	0	2	1	2	0
Total	262	61	15	3	98	2	14

### Differences in the levels of lipid molecules among feeding conditions

**Figure 4:**
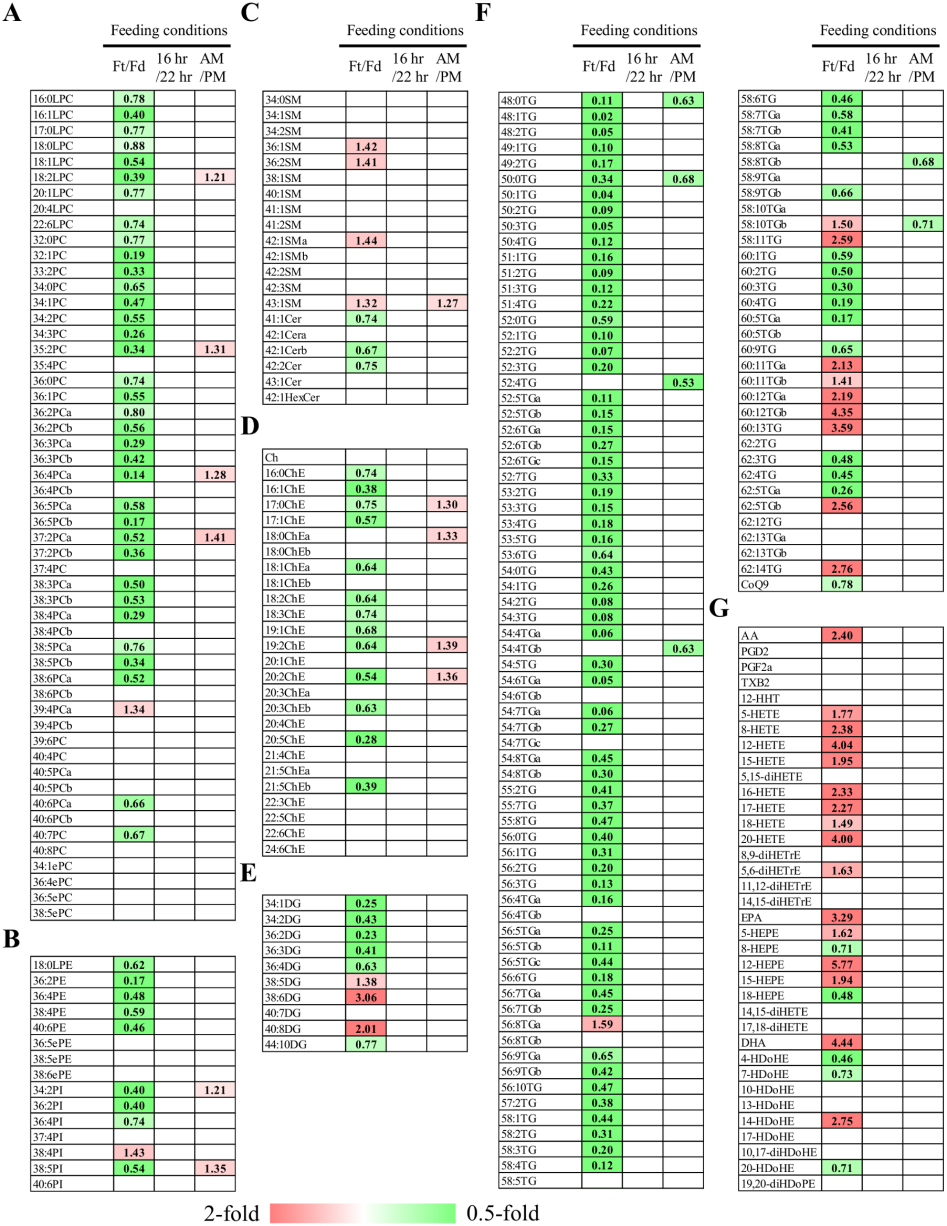
Heat maps of lipid molecules among rats subjected to different feeding conditions. The heat maps were generated with statistically significant different lipid molecules (p<0.05) of lysophosphatidylcholines and phosphoatidylcholines (A), other phospholipids (B), sphingolipids (C), cholesterol/cholesterolesters (D), diacylglycerols (E), triacylglycerol and coenzyme Q9 (F), and arachidonic acid, eicosapentaenoic acid, docosahexaenoic acid, and their metabolites (G). The numbers indicate the fold change of levels of individual lipid molecules in fasted over fed male rats (Ft/Fd), 16-hr fasted over 22-hr fasted male rats (16 vs. 22 hr), and blood collected from male rats at 10 AM over at 4 PM (AM/PM). The alphabet of the lipid class is used to distinguish 2 lipid species possessing the same formula. LPC, lysophosphatidylcholine; PC, phosphatidylcholine; ePC, ether-type PC; LPE, lysophosphatidylethanolamine; PE, phosphatidylethanolamine; ePE, ether-type PE; SM, sphingomyelin; Cer, ceramide; HexCer, hexosylceramide; Ch, cholesterol; ChE, cholesterolester; DG, diacylglycerol; TG, triacylglycerol; CoQ9, coenzyme Q9; AA, arachidonic acid; PG, prostaglandin; TX, thromboxane; HETE, hydroxyeicosatetraenoic aicd; diHETE, dihydroxyeicosatetraenoic acid; diHETrE, dihydroxyeicosatrienoic acid; EPA, eicosapentaenoic acid; HEPE, hydroxyeicosapentaenoic acid; DHA, docosahexaenoic acid; HDoHE, hydroxydocosahexaenoic acid; diHDoHE, dihydroxydocosahexaenoic acid; diHDoPE, dihydroxydocosapentaenoic acid.

To gain insight into the effects of feeding conditions on plasma lipid molecules, we next compared the levels of individual lipid molecules between rats that were fed and fasted, 16-hr fasted and 22-hr fasted, and from which blood was collected at 10 AM and 4 PM. The results of the comparative analysis showed that 183 lipid molecules were significantly different between fed and fasted male rats (Table 4). More than half of the lipid molecules in phospholipids, neutral lipids, and PUFAs and their metabolites were significantly different between fed and fasted male rats. Forty-four out of 46 significantly different phospholipids and 96 out of 109 significantly different neutral lipids were higher in fed rats than in fasted rats (Table 4). As shown in the heat map presented in Fig. 4E and F, levels of relatively longer and highly unsaturated DGs and TGs, such as 40∶8 DG, 58∶11, 60∶13, and 62∶14 TGs, were higher in fasted rats than in fed rats. On the other hand, 16 out of 21 significantly different PUFAs and their metabolites were higher in fasted rats than in fed rats (Fig. 4G). In contrast to the other lipid classes, only 7 out of 20 sphingolipids were statistically significant and their fold changes were less than 50% (Fig. 4C). No significantly different level of lipid molecules was observed between 16 hr-fasted and 22 hr-fasted male rats (Fig. 4). In addition, only 17 lipid molecules, such as 18∶2 LPC and 35∶2 PC, showed significantly different levels among different blood collection conditions (10 AM vs. 4 PM; Fig. 4).

**Table 4:** Differences in the number of lipid molecules at significantly higher levels among rats subjected to different feeding conditions.

		Fasting		Duration of fasting		Blood collection time	
Lipid classes	Total lipidmolecules	Fasted	Fed	16 hr	22 hr	10 AM	4 PM
Phospholipids	68	2	44	0	0	6	0
Sphingolipids	20	4	3	0	0	1	0
Neutral lipids	138	13	96	0	0	4	6
PUFAs and metabolites	36	16	5	0	0	0	0
Total	262	35	148	0	0	11	6

## Discussion

In the present study, we compared effects of multiple factors on the global profiles of lipid molecules in rat plasma. Based on overall comparison by OPLS-DA, feeding condition has a predominant role in the global profiles of lipid molecules, followed by sex and age. The number of statistically different levels in the lipid molecules between fasted and fed male rats reached 70% of the total lipid molecules determined, indicating a massive impact of feeding on lipid molecule levels. Sex-based differences in the levels of 42% of the lipid molecules tested were observed at 10 weeks of age and in 54% of the lipid molecules tested, at 30 weeks of age; whereas, age led to differences in the levels of 29% of the lipid molecules tested in male rats and 39% of lipid molecules in female rats. In contrast, overall comparison did not show any component contributing to separation among length of fasting period (16 hr vs. 22 hr) or diurnal time of sample collection (10 AM vs. 4 PM). In addition, there was no statistically significant difference in the levels of lipid molecules among the length of fasting period. Only 6.5% of the total lipid molecules determined showed significantly different levels among diurnal time of sample collection. These observations indicate that feeding or fasting condition, sex, and age are dominant factors influencing the global profiles of lipid molecules in rat plasma, while length of fasting period and diurnal time of sample collection has no or minor effects.

Sexual differences in hepatic gene expression, including cytochrome P450s, are well known in rodents [21], [22]. In the present study, we demonstrated that profiles of lipid molecules in plasma also sexually vary in rats. Levels of SMs and PUFAs containing ChEs were higher in both 10- and 30-week-old female rats. In humans, the levels of SM are higher in female than in male subjects throughout their lifespan, suggesting the sex-based differences in the plasma levels of SMs are conserved among these species [12], [14], [23]. The reason for higher levels of SMs in female rats has been proposed to be due to the nutritional preferences of women, because there are strong associations between fruit and vegetable intake and the serum levels of d18∶1/26∶1 SM [23]. In the present study, however, we housed rats with same diet between male and female. Thus, in addition to nutritional preference, there could be alternative factors responsible for the sexually different levels of SMs in plasma. On the other hand, the present study demonstrated that cholesterol and PUFAs were present in higher levels in female than in male rats, although the difference in the levels of most PUFAs levels in 10-week-old rats was not significant. In addition, the expression levels of hepatic acyl-coenzyme A:cholesterol transferase 2 were approximately 3-fold higher in female than in male subjects [24]. Thus, the higher levels of PUFA-containing ChEs in female rats might be due to higher levels of their substrates, as well as their synthesizing enzymes.

Unlike SMs and PUFA-containing ChEs, the levels of EPA, DHA, and their metabolites were higher in 30-week-old female but not in 10-week-old female rats. It remains unclear why the higher levels of EPA, DHA, and their metabolites in female rats were specific for the 30-week-old age group. However, in a previous study, the serum estradiol level in female rats was elevated from an undetectable level at 3 months of age, to 90 pM at 18 months of age [25]. Estrogens have been demonstrated to induce the synthesis of DHA in rat [26]. Thus, one possible mechanism for higher levels of PUFAs and their metabolites in 30-week-old female rats is the estrogen effect.

In the present study, we employed 10-week-old (young adult) and 30-week-old (matured adult) rats for examining the profiles of lipid molecules in plasma. Our study demonstrated that levels of relatively shorter and less unsaturated TGs (48∶0, 50∶0, and 52∶1 TGs) were higher in 30-week-old female rats than in 10-week-old rats, but that levels of relatively longer and highly unsaturated TGs (56∶10, 58∶11, and 60∶13 TGs) were not higher. It has been reported that blood TG levels increase between 10 weeks and 30 weeks in rats and that this increase is much more drastic in female than male rats [27]. Thus, relatively shorter and less unsaturated TGs dominantly contribute to age-associated increases in TGs observed in female rats, but the physiological mechanism for this remains unclear. However, it has been reported that estrogen treatment in turkey liver resulted in increased levels of TGs with total carbon numbers of 53, but in decreased levels of TGs with total carbon numbers of 57 [28]. Thus, estrogen might regulate the synthetic ratio of acyl side chains in TGs through an unknown mechanism in the liver, possibly leading to an increase in shorter, less unsaturated TGs in female plasma.

We have demonstrated that feeding and fasting condition represented a major contribution on the profiles of lipid molecules in plasma. The majority of lipid molecules that are significantly different between fasted and fed rats are food derivatives, which are at lower levels in fasted rats. On the other hand, relatively longer and highly unsaturated DGs and TGs, such as 40∶8 DG, 58∶11, 60∶13, and 62∶14 TGs, increased in fasted rats. In addition, PUFAs also increased in fasted rats. Because DGs and TGs are synthesized from glycerol and fatty acids, increased levels of PUFAs might contribute to increased levels of relatively longer and highly unsaturated DGs and TGs.

Unlike other lipid classes, sphingolipids were relatively stable among fasted and fed rats. Feeding condition, as well as food preference, is major concern of clinical tests. Recently, sphingolipids have emerged as biomarker candidates for cardiovascular events, traumatic brain injury, and depression [29]–[31]. Thus, stability of the levels of sphingolipids throughout feeding and fasting might be advantageous in the application of sphingolipids as biomarkers in clinical tests, although, the effect of gender on the levels of sphingolipids should be considered.

In conclusion, we demonstrated the effects of multiple factors on lipidomic profiles in rat plasma. Our results demonstrated that feeding condition and subjects’ sex and age are dominant factors modulating the levels of lipid molecules in rat plasma. The levels of most sphingolipids and PUFAs and their metabolites are sexually different. Age of female rats modulates differently the levels of relatively shorter and less unsaturated TGs and of relatively longer and highly unsaturated TGs. In addition, feeding and fasting condition also bi-directionally influenced the levels of relatively shorter and less unsaturated DGs and TGs and of relatively longer and highly unsaturated DGs and TGs. The effects of feeding condition are relatively smaller on the levels of sphingolipids than phospholipids, neutral lipids, and PUFAs and their metabolites. Taken together, our present study provides useful, fundamental information for exploring and validating lipid biomarkers in future preclinical studies and may also help to establish the regulatory standards for these studies.

## Supporting Information

File S1 **Supplemental tables. Table S1.** Nutrient composition of CRF-1 in 100 g. **Table S2.** Sample information. **Table S3.** Identified lipid molecules in plasma. **Table S4.** Normalized levels of lipid molecules in each sample group.
(XLSX)
